# Mixture design optimization of salvianolic acid B, tanshinone IIA, butein, and formononetin from *Salvia miltiorrhiza* and *Dalbergia odorifera* for myocardial infarction

**DOI:** 10.3389/fphar.2026.1783969

**Published:** 2026-06-25

**Authors:** Weihong Li, Kedi Liu, Chengzhao Liu, Qi Dai, Hong Yao, Hefei Wang, Ziheng Ding, Xinyan Cao, Peifeng Wei, Miaomiao Xi, Shengyong Zhang

**Affiliations:** 1 College of Life Sciences, Northwest University, Xi’an, China; 2 College of Pharmacy, Shaanxi University of Chinese Medicine, Xianyang, China; 3 TANK Medicinal Biology Institute of Xi’an, Xi’an, China; 4 State Key Laboratory of Flexible Electronics (LOFE) & Institute of Flexible Electronics (IFE), Northwestern Polytechnical University, Xi’an, China; 5 National Drug Clinical Trial Institute, The Second Affiliated Hospital, Shaanxi University of Chinese Medicine, Xixian New Area, China; 6 School of Pharmacy, Air Force Medical University, Xi‘an, China

**Keywords:** Salvia miltiorrhiza, Dalbergia odorifera, myocardial infarction, mixture design, optimal proportion

## Abstract

**Background:**

*Salvia miltiorrhiza-Dalbergia odorifera* (SM-DO) is a commonly used herb pair for treating cardiovascular diseases. It can treat myocardial infarction (MI) by regulating glycolysis and angiogenesis, but the optimal proportion of its active components remains unclear.

**Materials:**

Based on the enhanced simplex-centroid mixture design using Minitab 19 software, 19 proportions of salvianolic acid B (Sal-B), butein (But), tanshinone IIA (Tan IIA), and formononetin (For) were applied to an oxygen-glucose deprivation (OGD)-treated human cardiac microvascular endothelial cells (HCMVECs)-AC16 cells co-culture system. The proportions were independent variables, and viability, lactic acid, vascular endothelial growth factor (VEGF) of HCMVECs, and cardiac troponin I (cTnI) of AC16 cells were dependent variables. Regression equations were fitted and multi-objective optimization was performed to determine the OP, and experimental verification was conducted.

**Results:**

Stepwise regression analysis yielded regression equations for the four response indicators. The *R* ^2^ and adjusted *R* ^2^ of each model were at reasonable levels, and the predicted *R* ^2^ showed no significant decay. Multi-objective simultaneous optimization revealed OP of 60% Sal-B, 5% But, 5% Tan IIA, and 30% For. Experimental validation results showed that OP could promote lactic acid and VEGF production in OGD HCMVECs, enhance HCMVEC viability, and inhibit cTnI release in OGD AC16 cells. *In vivo*, OP increased levels of lactic acid, total protein lactylation (Pan-Kla), and VEGF in the myocardium of MI mice, and decreased levels of cTnI, creatine kinase-myocardial band (CK-MB), and myoglobin (Myo) in serum, alleviated pathological injury and fibrosis in myocardial tissues, and enhanced cardiac function in mice.

**Conclusion:**

This study preliminarily clarifies the OP composed of Sal-B, But, Tan IIA, and For that improves glycolysis and angiogenesis-related indicators and exerts cardioprotective effects, providing a new strategy for studies on active component formulations of traditional Chinese medicines. It is expected to lay a methodological foundation for the clinical application of SM-DO and the development of new drugs.

## 1 Introduction

Myocardial infarction (MI) is one of the major causes of death in coronary heart disease, which refers to atherosclerotic stenosis of coronary arteries induced by risk factors such as smoking, obesity, diabetes mellitus, hypertension, and hyperlipidemia, followed by rupture of plaques and formation of thrombi blocking the lumen of coronary arteries, resulting in a sharp decrease in or continuous interruption of the coronary blood supply, and ultimately causing acute ischemic injury to a part of the myocardium (Thygesen et al., 2018; Mensah et al., 2023). At present, the main treatments for MI patients are drug thrombolysis, percutaneous coronary intervention, and coronary artery bypass grafting, which can rapidly rebuild the coronary blood supply, slow the expansion of the infarct area and greatly improve the survival rate of patients (Reed et al., 2017). However, the microcirculation damage in the infarct margin caused by coronary artery occlusion inevitably results in insufficient myocardial blood perfusion, which restricts the transportation of oxygen, nutrients, and metabolic waste, thus leading to myocardial damage (Abbate et al., 2008). Therapeutic angiogenesis is an important strategy for MI treatment and improvement of cardiac function by promoting the proliferation, migration, and tube formation of cardiac microvascular endothelial cells, constructing collateral circulation of ischemic tissues, and restoring nutrition and the oxygen supply in the infarcted area to save dying myocardium (Li et al., 2022). Glycolysis is the primary metabolic energy source for endothelial cells (Pasut et al., 2025). Therefore, targeting endothelial cell glycolysis and angiogenesis may represent a potential strategy for the treatment of MI.

According to traditional Chinese medicine (TCM), MI is “chest paralysis and cardiac pain” caused by Qi stagnation and blood stasis. *Salviae miltiorrhizae*-*Dalbergiae odorifera* (SM-DO) is a classic herb pair for improving cardiovascular health. SM, as a monarch drug, focuses on circulating blood and removing blood stasis, whereas DO, as a minister drug, focuses on regulating Qi and alleviating pain, and the combination of the two drugs plays a role in treating MI. The active components of SM-DO, such as salvianolic acid B (Sal-B), (He et al., 2023), tanshinone IIA (Tan IIA) (Yang et al., 2023a), butein (But) (Zhang et al., 2011; Duan et al., 2017), and formononetin (For) (Qian et al., 2024; He et al., 2024; Yang et al., 2023b; Zhou et al., 2022), can exert cardioprotective effects by regulating energy metabolism, angiogenesis, inflammation, and autophagy. Significant breakthroughs have been made in researching the active components and therapeutic mechanisms of TCMs. However, the single-component-oriented research strategy inherently focuses on single targets. This approach deviates from TCM’s core theory of synergistic regulation, which emphasizes a ‘multi-component, multi-target, multi-biological processes’ network. Meanwhile, such a reductionist strategy fails to align with the complex pathological network in MI, which is governed by interactive regulation of “multi-gene, multi-protein, multi-biological processes”, thereby resulting in limited therapeutic efficacy. With respect to the strategy of multi-component compounding, the determination of component proportions lacks a systematic methodological framework. Consequently, it is difficult to quantitatively analyze the dose-effect relationships and synergistic mechanisms among the components.

To address the above issue, an experimental design method specifically suitable for proportion optimization studies is required. In conventional designs (such as factorial, orthogonal, or uniform designs), the levels of each factor can be varied independently. However, in the study of component proportions, the sum of the proportions of all components must be 100%. Changing the proportion of one component inevitably leads to corresponding changes in the proportions of the other components. Therefore, conventional designs cannot satisfy this constraint and may even generate unrealistic experimental points (e.g., the sum of component proportions exceeding 100%). In contrast, the mixture design involves the rational selection of a limited number of test points to make different proportions of each component to obtain a response model under a fixed total amount of mixture. Researchers can use the model to predict the optimal component proportions and analyze the individual and interactive effects of each component on response variables (Hare et al., 2024). This method has the characteristics of fewer experiments, high accuracy of parameter prediction, and simultaneous multi-objective optimization and is widely used in the fields of pharmacy (Patel et al., 2023), materials (Liu et al., 2023), the chemical industry (Teuschler et al., 2024), and food (Barnett et al., 2019). In a previous study, we used oxygen and glucose deprivation (OGD)-treated H9c2 cells and a mixture design to select 80% salvianolic acid A, 10% Sal-B, and 10% danshensu as the optimal proportion (OP) of the active components of SM for treating MI and verified its cardioprotective effect (Li et al., 2023). However, myocardial repair after MI is highly dependent on the complex interactions between vascular endothelial cells and cardiomyocytes, and simulating this critical process in a single-cell model is difficult. To more closely match the real microenvironment *in vivo*, this study constructed a human cardiac microvascular endothelial cells (HCMVECs)-AC16 cells co-culture system and introduced DO, which is a classic compatibility of SM in treating cardiovascular diseases, expanding the research components from water-soluble phenolic acid to fat-soluble components (Tan IIA, But, and For), aiming to screen the OP composed of Sal-B, But, Tan IIA, and For for treating MI through the regulation of endothelial glycolysis and angiogenesis *via* mixture design.

Therefore, this study established an OGD-treated co-culture model of HCMVECs and human cardiomyocyte AC16 cells. The viability, lactic acid, and vascular endothelial growth factor (VEGF) levels of HCMVECs and the cardiac troponin I (cTnI) level of AC16 cells are used as response indicators, and a mixture design was adopted to screen the OP composed of Sal-B, But, Tan IIA, and For, and verify its cardioprotective effects. It is expected to provide new ideas for the screening of pharmacologically active components and formula research of TCMs, and lay a foundation for the international promotion and secondary development of SM-DO and its formulations.

## 2 Materials and methods

### 2.1 Plant materials

All plant species were validated taxonomically using the Medicinal Plant Names Services (MPNS, http://mpns.kew.org) and Plants of the World Online (http://www.plantsoftheworldonline.org). The full taxonomic details are as follows:

*Salvia miltiorrhiza* Bunge [Lamiaceae; Salviae miltiorrhizae radix et rhizoma] (Chinese Pharmacopoeia). *Dalbergia odorifera* T. Chen [Fabaceae; Dalbergiae odoriferae lignum] (Chinese Pharmacopoeia).

### 2.2 Reagents and instruments

AC16 cells were purchased from Wuhan Pricella Biotechnology Co., Ltd, Wuhan, China. HCMVECs were purchased from Shanghai Tongwei Biotechnology Co., Ltd, Shanghai, China. Tan IIA (purity ≥98%; LOT: HR2185W6) was purchased from Baoji Chen Guang Biotechnology Co., Ltd, Baoji, China. Sal-B (purity ≥98%; LOT: nkl-00209250317) was purchased from Chengdu Nakeli Biological Technology Co., Ltd, Chengdu, China. For (purity ≥98%; LOT: YR-M0035240505) and But (purity ≥98%; LOT: YR-Q001240722) were purchased from Baoji Earay Biotechnology Co., Ltd, Baoji, China. DMEM/F12 culture medium (LOT: GP24090030695) was purchased from Wuhan Servicebio Biotechnology Co., Ltd, Wuhan, China. Fetal bovine serum (LOT: 2023057) was purchased from Biological Industries Israel Beit-Haemek Ltd., Beit Haemek, Israel. Trypsin-EDTA solution (LOT: 240004007) was purchased from Beijing Solarbio Science and Technology Co., Ltd, Beijing, China. Penicillin‒streptomycin mixture (LOT: 24CN0300) was purchased from Saiwen Innovation (Beijing) Biological Technology Co., Ltd, Beijing, China. The CellTiter-Glo 2.0 Assay was purchased from Promega (Beijing) Biotech Co., Ltd, Beijing, China. cTnI (LOT: 202503) and VEGF (LOT: 202503) ELISA kits were purchased from Shanghai Enzyme-linked Biotechnology Co., Ltd, Shanghai, China. BCA (LOT: P1004) protein quantitative kit was purchased from Shaanxi Zhonghui Hecai Biomedical Technology Co., Ltd, Xi’an, China. Anti-L-Lactyl Lysine Rabbit mAb (LOT: RO041906) was purchased from PTM Biolab Inc., Hangzhou, China. A CO_2_ cell incubator (371) was purchased from Thermo Fisher Scientific Inc., Waltham, USA. An inverted microscope (CKX53) was purchased from Olympus (China) Co., Ltd, Beijing, China. A benchtop large-capacity high-speed refrigerated centrifuge (H2050R) was purchased from Hunan Xiangyi Laboratory Instrument Development Co., Ltd, Changsha, China. An analytical balance (BSA124S) was purchased from Sartorius AG, Göttingen, Germany. A microplate reader (MR-96A) was purchased from Shenzhen Mindray Biomedical Electronics Co., Ltd, Shenzhen, China. A multimode plate reader (VICTOR X2) was purchased from PerkinElmer, Inc., Waltham, USA.

### 2.3 Preparation of drug-containing serum

#### 2.3.1 Animal grouping and treatment

Sprague–Dawley rats (SPF grade, 7 weeks old, body weight 200–250 g) were purchased from Xi’an Jiaotong University (Animal Production License No. SCXK (Shaan) 2023-002). The rats were housed individually under strictly controlled environmental conditions: 22 °C ± 2 °C, 55%–75% humidity, and a 12-h light/dark cycle. The experimental protocol was approved by the Animal Ethics Committee of Shaanxi University of Chinese Medicine (Approval No. SUCMDL20250617002).

Using a random number table, the 12 rats were divided into two groups: the blank control (BC) group and the SM-DO group. All the groups received intragastric administration twice daily for 7 consecutive days. One and a half hours after the final treatment, the rats were deeply anesthetized by an intraperitoneal injection of 20% urethane (0.75 g/kg), and blood samples were subsequently collected from the abdominal aorta. The treatments were as follows.

1. SM-DO group: SM (1.58 g/kg) and DO (0.79 g/kg) formula granules.
2. BC group: Sterilized purified water (20 mL/kg; equivalent volume).

#### 2.3.2 Serum processing

After standing for 1 h, the blood was centrifuged at 3000 rpm for 10 min. The serum was aseptically separated in a biosafety cabinet, followed by inactivation in a 56 °C water bath for 30 min. Sera from the same group were pooled, filtered through a 0.22 μm membrane for sterilization, aliquoted into 1.5 mL centrifuge tubes, and stored at −80 °C until use.

### 2.4 Cell Co-culture and OGD treatment

AC16 cells were cultured in DMEM/F12 containing 10% fetal bovine serum and 1% penicillin‒streptomycin in a CO_2_ incubator (37 °C, 5% CO_2_). When the cells reached 80% confluence, the culture medium was discarded, the cells were washed with PBS three times, 2 mL of trypsin (0.25%) was added, and the cells were incubated for approximately 2 min. When the cells became round and fell off, 2 mL of DMEM/F12 was immediately added to terminate the incubation. The adherent cells were detached into a suspension with a pipette and transferred to a 15 mL centrifuge tube. After centrifugation at 1000 *g* for 5 min, the cells were transferred to a new cell culture bottle at a 1:2 passage ratio and placed in a CO_2_ incubator. The DMEM/F12 was changed every 48 h. HCMVECs were cultured in specialized medium, and the procedure was the same as that for AC16 cells.

HCMVECs and AC16 cells in the logarithmic growth phase were digested and collected, and then seeded into the upper and lower layers of a Transwell chamber at a cell number ratio of 3:1 (HCMVECs: AC16). The upper layer was cultured with HCMVECs-specific medium, and the lower layer was cultured with DMEM/F12 complete medium. The Transwell chamber was placed in a cell culture incubator overnight to allow cell attachment and establish the co-culture system.

When the cells reached 80% confluence, the original complete medium was discarded, and the cells were gently washed twice with pre-warmed PBS. Then, the medium was replaced with sugar-free medium, and the cells were quickly transferred to a 37 °C anaerobic incubator (Shanghai Yuejin Medical Instruments Co., Ltd., model: YQX-II). The incubator was continuously infused with a mixed gas (10% H_2_, 5% CO_2_, 85% N_2_) to establish a hypoxic environment. Hydrogen gas reacted with residual oxygen in the chamber under the action of a palladium catalyst to generate water, thereby ensuring and maintaining a strictly anaerobic state. The oxygen concentration inside the chamber was monitored in real time using an oxygen detector (Dongguan Wanchuang Electronic Products Co., Ltd., model: AC8100). The OGD treatment lasted for 8 h.

### 2.5 CCK-8 method for screening optimal drug concentrations

When the cells reached 80% confluence, they were inoculated in 96-well plates at a density of 1 × 10^4^ cells/well and incubated overnight. The culture medium was replaced with drug-containing glucose-free culture medium and then placed in an anaerobic incubator for 8 h. After OGD-treated, 10 μL of CCK-8 solution was added to each well, and the absorbance was measured at 450 nm after 2 h of incubation.

### 2.6 Experimental points in mixture design

According to the optimal concentrations of Sal-B, But, Tan IIA, and For determined in Section 2.5, the four compounds were dissolved in dimethyl sulfoxide (DMSO) to prepare stock solutions at 1000-fold of their respective optimal concentrations. The stock solutions were aliquoted for single-use and stored at −80 °C. The final DMSO concentration in the working solution was 0.1% (v/v). Preliminary experiments confirmed no interference from the solvent system on cell viability. Before use, the stock solutions were diluted with sugar-free medium to their respective optimal working concentrations and prepared fresh.

An augmented simple centroid mixture design was used, and 19 mixture proportion schemes were generated by Minitab software, with the volume percentage of each component in the mixture ranging from 0% to 100%. The working solutions of the four components were mixed according to the volume ratios shown in each scheme and then added to the upper and lower chambers of the Transwell system.

### 2.7 Cell Titer-Glo® luminescent (CTG) assay for determining cell viability

After OGD-treated, 100 μL and 500 µL of CellTiter-Glo 2.0 Assay Reagent were added to the upper and lower chambers of each Transwell, respectively, and the contents were mixed for 2 min with an orbital oscillator and incubated for 10 min at room temperature. 100µL of the liquid in the upper and lower chambers was removed, transferred to a 96-well plate with a white wall and a transparent bottom, and the luminescence signal was read with a multimode plate reader. Cell viability (%) = (RLUexperimental − RLUblank)/(RLUcontrol − RLUblank) × 100%.

### 2.8 MI model in C57BL/6 mice

SPF-grade male C57BL/6 mice (7 weeks old, 23–25 g) were purchased from Xi’an Jiaotong University (Animal Production License No. SCXK (Shaan) 2023-002) and housed under controlled conditions. The mice were anesthetized *via* inhalation of 2% isoflurane and secured in a supine position. Following disinfection, a 1 cm incision was made along the left sternal border to expose the heart. The left anterior descending coronary artery was ligated approximately 3 mm from its origin, with successful model induction confirmed by immediate whitening of the anterior left ventricular wall. Sham-operated mice underwent the same surgical procedure but without coronary artery ligation. The protocol was approved by the Animal Ethics Committee (SUCMD120240306011).

### 2.9 Animal grouping and drug administration

Mice were randomly assigned to groups using a random number table and received corresponding treatments. Group allocation information was generated and sealed by personnel not involved in subsequent experiments. The OP dosage was converted from the optimal proportion obtained in the co-culture screening to a mass ratio and determined through preliminary experiments. OP was dissolved in DMSO and Tween-80, then diluted with sterile water to the high dose (final solvent concentration: 10% DMSO +5% Tween-80); the medium and low doses were obtained by further dilution of the high-dose solution. Preliminary experiments showed no significant difference in myocardial enzyme indicators between the MI + solvent group and the MI group, indicating that the solvent system did not interfere with the experimental results. The doses for the SM-DO and Meto groups were calculated by converting clinical human doses to animal equivalents. All groups received intragastric administration once daily for 28 consecutive days.

1. Sham group: Underwent threading without ligation and received sterile water (10 mL/kg);
2. MI group: Underwent ligation and received sterile water (10 mL/kg);
3. OP low-dose group: Underwent ligation and received 25 mg/kg OP *via* gavage.
4. OP medium-dose group: Underwent ligation and received 50 mg/kg OP *via* gavage.
5. OP high-dose group: Underwent ligation and received 100 mg/kg OP *via* gavage.
6. SM-DO group: Underwent ligation and received SM (1.58 g/kg) and DO (0.79 g/kg) formula granules *via* gavage.
7. Metoprolol (Meto) group: Underwent ligation and received 15 mg/kg metoprolol *via* gavage.

All examinations were performed by blinded researchers, with group information concealed during data collection and analysis.

### 2.10 Echocardiographic assessment of cardiac function in mice

Cardiac function was assessed using a VEVO 2100 ultrasound imaging system. Key parameters were analyzed, including left ventricular end-systolic diameter (LVESD), left ventricular end-diastolic diameter (LVEDD), left ventricular fractional shortening (LVFS), left ventricular end-systolic volume (LVESV), ejection fraction (EF), and fractional shortening (FS) to evaluate changes in cardiac performance.

### 2.11 HE and masson staining for detection of myocardial injury and collagen deposition

Heart tissues were rinsed with pre-cooled 1× PBS to remove blood, fixed in 4% paraformaldehyde, dehydrated, embedded in paraffin, and sectioned coronally into 5 μm slices. Morphological changes and myocardial injury were observed under a microscope following Hematoxylin and Eosin (HE) staining, while collagen deposition and fibrosis were evaluated using Masson staining.

### 2.12 Enzymatic assay for lactic acid level

The HCMVECs culture medium was discarded, and the cells were washed once with ice-cold PBS. Pre-cold RIPA lysis buffer (containing 1% protease inhibitor and 1% phosphatase inhibitor) was added, and the cells were lysed on ice for 30 min. The lysate was centrifuged at 12000 *g* for 15 min at 4 °C, and the supernatant was collected as the total protein extract. The protein concentration was determined using a bicinchoninic acid (BCA) protein assay kit, and the samples were aliquoted and stored at −80 °C.

Mouse myocardial tissues were rinsed with ice-cold PBS to remove blood, then homogenized. Pre-cold RIPA lysis buffer (containing 1% protease inhibitor, 1% phosphatase inhibitor, and 1% deacetylase inhibitor) was added. Subsequent steps were the same as those for the cell samples.

Lactic acid concentration was measured according to the manufacturer’s instructions of the lactic acid assay kit. The absorbance was read at the appropriate wavelength, and the final concentration was calculated.

### 2.13 Enzyme-linked immunosorbent assay for VEGF, cTnI, CK-MB and MYO levels

VEGF levels in HCMVECs and mouse myocardial tissue were measured using a VEGF ELISA kit according to the manufacturer’s instructions. cTnI levels in AC16 cells culture supernatant were determined using a cTnI ELISA kit. Mouse serum levels of cTnI, CK-MB, and Myo were measured using the corresponding ELISA kits. The absorbance was read at the appropriate wavelength, and final concentrations were calculated.

### 2.14 Detection of Pan-Kla levels by western blot

Mouse myocardial tissue protein was extracted as described in Section 2.11. The protein supernatant was mixed with loading buffer, heated at 100 °C for 10 min to denature the protein, and then separated by 10% SDS-PAGE. After electrophoresis, the gel containing the protein samples was wet-transferred onto a PVDF membrane. The membrane was blocked with 5% non-fat milk at 25 °C for 2 h, followed by overnight incubation at 4 °C with primary antibodies against Pan-Kla (1:1000) and β-actin (1:1000). The membrane was then washed three times (10 min each) with PBS containing 0.1% Tween-20 (PBST) and incubated with secondary antibody at 37 °C for 2 h. After three additional 10-min washes with PBST, protein bands were visualized using an enhanced chemiluminescence (ECL) kit.

### 2.15 Statistical analysis

#### 2.15.1 Sample size and statistical software

The sample size was determined based on common practice in similar experimental designs in the field (n = 6 per group for animal experiments (Luo et al., 2025; Chen et al., 2025), n = 3 independent replicates for cell experiments (Wan et al., 2023; Wang et al., 2022) and the availability of research resources. For Transwell co-culture experiments, 6 independent replicates were performed to ensure the stability of multi-indicator detection in the complex system.

Minitab (ver. 19) was used to construct the mixture design. The model was fitted by stepwise regression (variable selection criterion: *P* ≤ 0.15), and OP was solved based on this model.

GraphPad Prism (ver. 9) was used for statistical analysis and graphing. Data are expressed as means ± SD. Normality was assessed using the Shapiro-Wilk test, and homogeneity of variances was assessed using the Brown-Forsythe test, combined with Q-Q plots and individual value plots for comprehensive evaluation. No violation of the assumptions for parametric tests was observed. Therefore, overall comparisons among groups were performed using one-way analysis of variance (ANOVA), followed by Tukey’s *post hoc* test for pairwise comparisons. A value of *P* < 0.05 was considered statistically significant.

#### 2.15.2 Model fitting and analysis

With viability, lactic acid, the VEGF level of HCMVECs, and the cTnI level of AC16 cells as dependent variables and the proportions of the four components as independent variables, the full quartic model was selected, stepwise regression was used to screen the terms included in the model, and the least squares method was used to estimate the unknown coefficients in the model. The fitted regression model was evaluated *via* a lack-of-fit test and statistics, including mainly *R* ^2^, adjusted *R* ^2^, and the predicted *R* ^2^.

#### 2.15.3 Multi-objective simultaneous optimization

In this study, higher viability, lactic acid, and VEGF levels in HCMVECs and lower cTnI level in AC16 cells indicated better drug treatment effects. To optimize the above variables at the same time, the lower limit of viability, lactic acid, and VEGF of HCMVECs was set as the minimum value of the OGD-treated group, the target value was 80% of the maximum value of the control group, and the upper limit was the maximum value of the control group. The lower limit of the cTnI level of the AC16 cells was set as the minimum value of the control group, the target value was the minimum value of the control group/80%, and the upper limit was the maximum value of the OGD-treated group. The multi-objective simultaneous optimization program of Minitab software was run to find the OP of Sal-B, But, Tan IIA, and For. Individual desirability (d) quantifies the satisfaction of a single variable, and composite desirability (D) comprehensively evaluates the overall satisfaction of all the variables, with agreeableness values ranging from 0 to 1. 1 represents the ideal situation, and 0 represents that one or more of the responses are out of the acceptable range.

The OP obtained above was used as the high dose, and was diluted 2-fold and 4-fold with sugar-free medium to obtain the medium and low doses, respectively.

## 3 Results

### 3.1 Establishment and validation of the mixture design model

**FIGURE 1:**
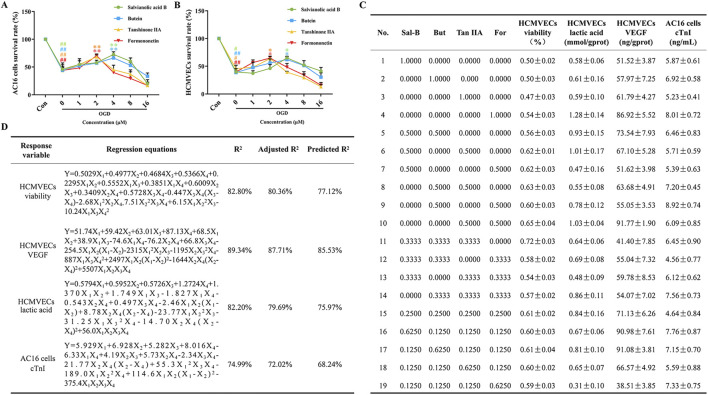
Mixture design and regression model fitting for salvianolic acid B (Sal-B), butein (But), tanshinone IIA (Tan IIA), and formononetin (For). **(A–B)** Effects of drug concentrations on viability of oxygen-glucose deprivation (OGD)-treated AC16 cells and human cardiac microvascular endothelial cells (HCMVECs) (means ± SD, n = 3). ^##^ *P* < 0.01, ^#^ *P* < 0.05 vs. Con; **P < 0.01, *P < 0.05 vs. OGD. **(C)** Mixture design and results of viability, vascular endothelial growth factor (VEGF), lactic acid levels of HCMVECs, and cardiac troponin I (cTnI) level of AC16 cells (means ± SD, n = 6). **(D)** Response models for the same variables.

As shown in Figures 1A,B, the viability of OGD-treated AC16 cells and HCMVECs was significantly decreased (*P* < 0.01) compared to the Control group. It reached a maximum at concentrations of 4 μM for Sal-B and But, and 2 μM for Tan IIA and For (*P* < 0.01).

The viability, VEGF, lactic acid levels of HCMVECs, and cTnI levels of AC16 cells were used as response indicators, and the experiments were conducted in 19 different proportions according to the mixture design; the results are shown in Figure 1C. The regression equations for the four response indicators were derived using stepwise regression analysis based on a complete quartic polynomial model. The global F-test for each model yielded *P*-values of less than 0.05, indicating statistical significance. Conversely, the lack-of-fit tests for all models resulted in *P*-values greater than 0.05, confirming no significant lack-of-fit. *R* ^2^ and adjusted *R* ^2^ were at reasonably high levels, with predicted *R* ^2^ showing no significant decay. These results collectively suggest that the models possess strong predictive ability without overfitting. The detailed results are presented in Figure 1D.

### 3.2 Analysis of fitted models, multi-objective optimization, and validation of optimal proportion

**FIGURE 2:**
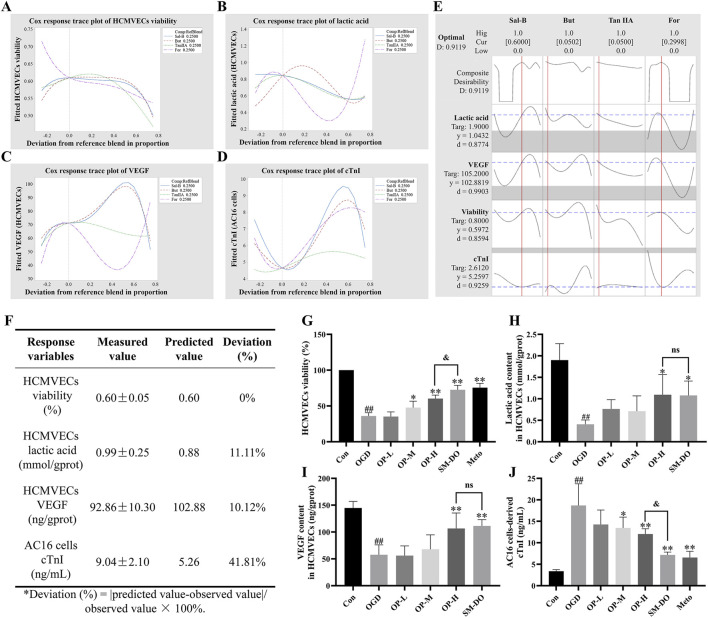
Analysis and validation of the optimal proportion (OP). **(A–D)** Cox response trace plots for viability, VEGF, lactic acid levels of HCMVECs, and cTnI level of AC16 cells. **(E)** Schematic of multi-objective optimization. **(F)** Validation results of the same variables (n = 6, means ± SD). **(G–J)** Efficacy comparison of OP vs. *Salvia miltiorrhiza*-*Dalbergia odorifera* (SM-DO). ^##^ *P* < 0.01 vs. Con; **P < 0.01, *P < 0.05 vs. OGD; ^&^ *P* < 0.05 vs. SM-DO.

Cox response trace plots were utilized to visualize the effects of varying the proportions of Sal-B, But, Tan IIA, and For on HCMVEC viability. As shown in Figure 2A, each curve represents the effect of changing one component’s proportion while holding the others constant. The intersection point of the curves represents the centroid (reference blend). Deviations in Sal-B or But proportions from the reference blend reduced viability, whereas decreasing Tan IIA also reduced viability but increasing it produced a biphasic effect (initial increase followed by a decrease). Viability was negatively correlated with the proportion of For it increased as the proportion of For decreased and *vice versa*. As shown in Supplementary Figure S1A, the optimal proportion (OP) for maximizing viability was predicted from the fitted model to be 40% Sal-B: 29% But: 31% Tan IIA. At this OP, viability was estimated to reach 72%, with a high desirability of 0.9508.

As shown in Figure 2B, relative to the reference blend, lactic acid levels exhibited a negative correlation with Sal-B proportion, increasing when the proportion was reduced and decreasing when the proportion was raised. For But, a decrease in its proportion reduced the lactic acid level, while an increase produced a biphasic effect that was characterized by an initial increase followed by a decrease. Any deviation of the Tan IIA proportion from the reference level resulted in decreased lactic acid. The proportion of For also showed a complex relationship. A decrease caused lactic acid levels to first increase and then decrease, while an increase produced the opposite trend. As shown in Supplementary Figure S1B, the OP for maximizing lactic acid levels was predicted by the fitted model to be 75% Sal-B: 25% For. At this OP, the lactic acid level was estimated to reach 1.8961 mmol/gprot, with a high desirability value of 0.9996.

As shown in Figure 2C, the VEGF level in HCMVECs showed a complex relationship with the proportions of Sal-B and But. A decrease in the proportion of either component relative to the reference blend led to a gradual decline in VEGF levels. Conversely, an increase in their proportions resulted in a biphasic response, characterized by an initial increase followed by a decrease. Furthermore, deviation in the proportion of Tan IIA from the reference blend resulted in reduced VEGF levels. A decrease in the proportion of For caused lactic acid levels to first increase and then decrease, while an increase produced the opposite trend. As shown in Supplementary Figure S1C, the OP for maximizing VEGF levels was predicted by the fitted model to be 25% Sal-B: 60% But: 15% For. At this OP, the VEGF level was estimated to reach 102.5199 ng/gprot, with a high desirability value of 0.9888.

As shown in Figure 2D, a decrease in the proportions of Sal-B, But, and For relative to the reference blend resulted in a gradual increase in cTnI levels. However, with increasing proportions of these components, the cTnI level exhibited a biphasic response, first increasing and then subsequently decreasing. A decrease in the proportion of Tan IIA relative to the reference blend led to a gradual decrease in cTnI levels. Conversely, an increase in the proportion of Tan IIA resulted in a biphasic response, characterized by an initial increase followed by a decrease. As shown in Supplementary Figure S1D, the OP for minimizing cTnI levels was predicted from the fitted model to be 77% Sal-B: 23% For. At this OP, the cTnI level was estimated to reach 3.2869 ng/mL, with a desirability value of 0.9828.

As shown in Figure 2E, when four response indicators (HCMVEC viability, lactic acid, VEGF level, and AC16 cell cTnI level) were optimized simultaneously, the OP was determined to be 60% Sal-B, 5% But, 5% Tan IIA, and 30% For, with a composite desirability of 0.9119. The predictive accuracy of these optimal proportions was validated through six independent experiments, as shown in Figure 2F. For all response indicators except cTnI, the measured values closely matched the predicted values, indicating that the model provides reliable predictions for these indicators. Although some discrepancy was observed between the predicted and measured cTnI values, the model successfully identified a formulation with clearly defined components and proportions that significantly reduced cTnI levels in OGD-treated AC16 cells.

Compared to the Control group, the OGD-treated HCMVECs exhibited significantly (*P* < 0.01) lower viability (Figure 2G), lactic acid (Figure 2H), and VEGF (Figure 2I) levels, while the OGD-treated AC16 cells showed a significantly (*P* < 0.01) higher cTnI (Figure 2J) level. Treatment with OP, SM-DO, or Meto significantly increased viability, lactic acid, and VEGF levels in OGD-treated HCMVECs and decreased the cTnI level in OGD-treated AC16 cells. Furthermore, the effects of OP on lactic acid and VEGF in HCMVECs were comparable to those of SM-DO. However, the effect of OP on increasing OGD-treated HCMVEC viability and decreasing cTnI in AC16 cells was significantly (*P* < 0.05) weaker than that of SM-DO.

### 3.3 OP improves cardiac function and alleviates myocardial injury in myocardial infarction mice

**FIGURE 3:**
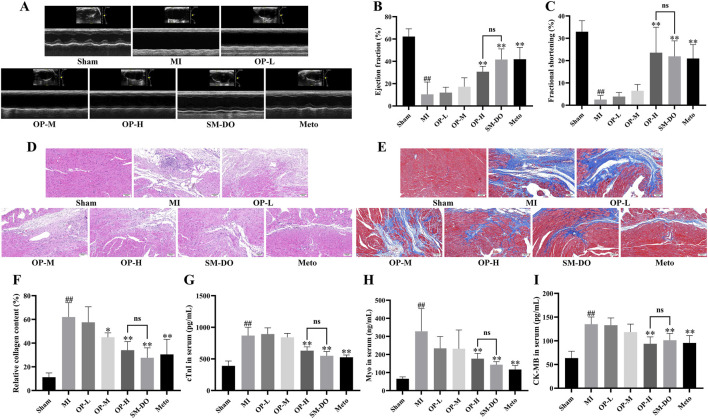
OP ameliorates cardiac function and pathology in myocardial infarction (MI) mice. **(A)** Representative echocardiograms. **(B)** Ejection fraction (EF) (means ± SD, n = 6). **(C)** Fractional shortening (FS) (means ± SD, n = 6). **(D)** Myocardial pathomorphology (HE staining, n = 6, scale bar: 100 μm). **(E)** Myocardial fibrosis (Masson staining, scale bar: 100 μm). **(F)** Quantitative analysis of myocardial fibrosis (means ± SD, n = 6). **(G–I)** Serum levels of cardiac troponin I (cTnI), myoglobin (Myo), and creatine kinase-MB (CK-MB) in myocardial infarction mice (means ± SD, n = 6). ##P < 0.01 vs. Sham; *P < 0.05, **P < 0.01 vs. MI.

As shown in Figures 3A–C, compared with the Sham group, the MI group exhibited significantly decreased EF and FS (*P* < 0.01), along with significantly increased LVESD, LVESV, and LVEDV (*P* < 0.01) (Supplementary Figures S2A-C). Treatment with OP-H, SM-DO, and Meto significantly reversed these changes by increasing EF and FS values and decreasing LVESD, LVEDV, and LVESV values compared with the MI group. Furthermore, no significant differences were observed in these indices between the OP-H and SM-DO groups.

Histopathological evaluation of myocardial tissue by HE staining is shown in Figure 3D. The Sham group exhibited well-aligned myocardial structure and normal cardiomyocyte morphology. In contrast, the MI group displayed severe myocardial pathology, including disorganized tissue architecture, blurred cell boundaries, nuclear dissolution, and interstitial vacuolization. Treatment with OP-L, OP-M, OP-H, SM-DO, and Meto ameliorated these injury phenotypes to varying degrees, manifested as improved myocardial structural integrity and reduced cellular dissolution and vacuolization.

Masson staining of myocardial tissue is presented in Figures 3E,F. The Sham group displayed well-aligned myocardial structure, predominantly red-stained cardiomyocytes with minimal blue-stained collagen fibers. In contrast, the MI group exhibited severe structural disorganization, evident tissue rupture, and a significant increase in blue collagen deposition (*P* < 0.01). Treatment with OP-M, OP-H, SM-DO, and Meto significantly reduced this collagen deposition, resulting in more ordered myocardial arrangement and diminished interstitial rupture. Furthermore, no significant difference in the anti-fibrotic effect was observed between the OP-H and SM-DO groups.

As shown in Figures 3G–I, the serum concentrations of cTnI, Myo, and CK-MB were significantly elevated (*P* < 0.01) in the MI group compared with the Sham group. Treatment with OP-H, SM-DO, and Meto significantly reduced the serum concentrations of these biomarkers compared with the MI group. No significant differences were observed in the effects on cTnI, Myo, and CK-MB between the OP-H and SM-DO groups.

### 3.4 OP improves glycolysis- and angiogenesis-related indicators in the infarcted myocardium

**FIGURE 4:**
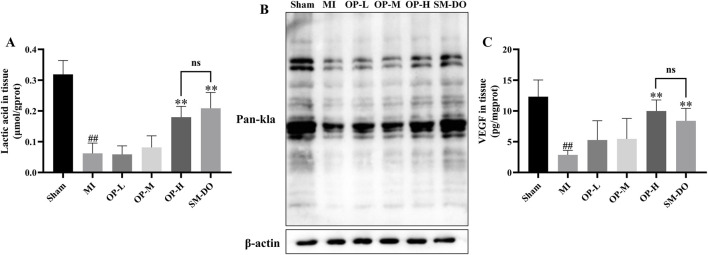
OP improves glycolysis and angiogenesis-related indicators in the myocardium of MI mice. **(A–C)** Myocardial levels of lactic acid **(A)**, pan-lysine lactylation (Pan-kla) expression **(B)**, and VEGF **(C)** (means ± SD, n = 6). ^##^ *P* < 0.01 vs. Sham; **P < 0.01 vs. MI.

As shown in Figures 4A,B, compared with the Sham group, the MI group exhibited significantly decreased myocardial lactic acid levels (*P* < 0.01), accompanied by a corresponding reduction in total protein lactylation. In contrast, both OP-H and SM-DO treatments significantly increased lactic acid levels (*P* < 0.01) and correspondingly elevated total protein lactylation compared with the MI group. Furthermore, no significant difference was observed in the effects on lactic acid levels between the OP-H and SM-DO groups.

As shown in Figure 4C, compared with the Sham group, the MI group exhibited significantly decreased myocardial VEGF levels (*P* < 0.01). Treatment with OP-H and SM-DO significantly increased VEGF levels compared with the MI group (*P* < 0.01). No significant difference was observed in the effect on VEGF levels between the OP-H and SM-DO groups.

## 4 Discussion

MI is a myocardial injury resulting from interruption of coronary artery blood flow and represents one of the most critical emergencies with the highest mortality rate among cardiovascular diseases (Boeddinghaus et al., 2025). SM-DO has a long history and proven efficacy in the treatment of MI. Sal-B, Tan IIA and But, For are important active components of SM and DO, respectively. However, the optimal proportion of these four components in regulating endothelial glycolysis, angiogenesis, and myocardial protection remains unclear. Therefore, this study utilized an HCMVECs-AC16 cell co-culture model and employed a mixture design strategy to screen the OP of 60% Sal-B, 5% But, 5% Tan IIA, and 30% For, with HCMVEC viability, lactic acid, VEGF, and AC16 cell cTnI levels as indicators, and verified its myocardial protective effects. The results showed that OP promoted lactic acid production, upregulated VEGF levels, enhanced HCMVEC viability, reduced cTnI release in AC16 cells, and alleviated myocardial cell injury.

This study is exploratory in nature, aiming to preliminarily screen key components and establish a proportion-response relationship. In mixture design, stepwise regression has been widely adopted for its computational simplicity and efficiency (Cornell, 2002; Harith et al., 2025; Cruz-Salgado et al., 2026; Chen et al., 2022). In this study, stepwise regression was performed using Minitab software (which maintains the hierarchical model structure by default), and independent experiments were conducted to validate the model-predicted results.

In mixture design regression modeling, *R* ^2^ serves as a key metric to evaluate the goodness of fit, quantifying the proportion of variance in the dependent variable that can be explained by the model. The adjusted *R* ^2^ is a correction to *R* ^2^, and its lack of significant attenuation compared with *R* ^2^ indicates that the model has strong explanatory power and no overfitting phenomenon. The predictive *R* ^2^ is used to evaluate the model’s predictive ability for new data. In this study, the *R* ^2^ and adjusted *R* ^2^ of each response model were at reasonably high levels, with no obvious attenuation in predictive *R* ^2^, and the lack-of-fit test *P*-values were all greater than 0.05, indicating that the models had adequate predictive ability and no apparent overfitting. While stepwise regression yields desirable fitting performance in this study, its inherent limitations cannot be overlooked. Penalized regression and other approaches can be considered for model comparison in subsequent studies to pursue better fitting results.

Cox response trace plots visualize the contribution of each component to the different variables. Regarding lactic acid production in HCMVECs, increasing the percentage of Sal-B in the mixture gradually reduced lactic acid levels, while Tan IIA promoted lactic acid production at low concentrations but inhibited it at high concentrations. This indicates that the effects of Sal-B and Tan IIA on glycolysis are dose-dependent. Regarding VEGF expression in HCMVECs, Sal-B, But, and Tan IIA all exhibited a similar biphasic effect, promoting VEGF at low proportions but inhibiting it at high proportions. This suggests that these three compounds have an optimal concentration range. With respect to the viability of HCMVECs, an increase in the proportion of For continuously reduced cell viability, indicating a dose-dependent inhibitory effect. Sal-B, But, and Tan IIA increased viability at low proportions but decreased it at high proportions, also suggesting that these three compounds have an optimal concentration range for regulating the viability of HCMVECs.

In recent years, successful and timely revascularization has significantly improved the survival rate and prognosis of MI patients. However, the “no-reflow” phenomenon caused by microcirculatory dysfunction still leads to persistent ischemia in the infarct border zone of some patients (Kaul et al., 2023). Therapeutic angiogenesis can improve blood flow perfusion in the ischemic myocardium and alleviate myocardial damage (Badimon and Borrell, 2018). Endothelial cells construct new microvascular networks through proliferation, migration, and tube formation (Zeng and Fu, 2025).

Under normal physiological conditions, the metabolism of endothelial cells is highly dependent on glycolysis. Unlike most cell types, less than 1% of pyruvate enters the tricarboxylic acid cycle in endothelial cells, and over 80% of ATP is derived from glycolysis. When endothelial cells proliferate or migrate, the glycolytic rate can double, and this metabolic profile has the advantages of low oxygen consumption and reduced reactive oxygen species production (Wong et al., 2017). The lactic acid level, as the end product of glycolysis, can reflect the glycolytic activity of endothelial cells. In recent years, lactylation, a novel post-translational modification driven by lactic acid, has been reported, and studies have shown that it participates in metabolic regulation and cardiac repair after MI (Zhang et al., 2019; Wang et al., 2022). Therefore, this study measured the total protein lactylation level in myocardial tissue. In this study, OP significantly increased the lactic acid level in OGD HCMVECs and significantly elevated the lactic acid and total protein lactylation (Pan-Kla) levels in the myocardial tissue of MI mice, suggesting that this formulation can improve glycolysis-related metabolic indicators. However, it should be noted that this study only measured total protein lactylation levels and did not perform quantitative analysis on histone-specific sites or specific target proteins. Therefore, the functional implications of lactylation and the epigenetic regulatory mechanisms cannot be determined, and further lactyl-proteomics analysis is needed in subsequent studies. Some scholars have proposed the “theory of limited representative components”, which means that through *in vivo* and *in vitro* pharmacological experiments, when there is no significant difference (*P* > 0.05) in the pharmacological effects between the combination of a limited number of active components and the whole component, the comprehensive properties of the representative component combination can represent the properties of the whole component (Xiujuan, 2016). The results revealed that the efficacy of OP was comparable to that of SM-DO in terms of lactic acid level, indicating that Sal-B, But, Tan IIA, and For are the representative components in SM-DO for increasing lactic acid levels. When only the lactic acid level was used as the response variable, the optimal proportion was 75% Sal-B:25% For, and the predicted lactic acid level could be restored to 1.8961 mmol/gprot, suggesting that Sal-B and For are the main components in OP for increasing lactic acid levels, which is consistent with the high proportion of Sal-B and For in OP.

VEGF is one of the key factors in the regulatory network of angiogenesis, which can promote endothelial cell proliferation, migration, and tube formation (Alissa et al., 2026). In this study, OP significantly increased the VEGF level in OGD HCMVECs and the myocardial VEGF level in MI mice. Moreover, the efficacy of OP was comparable to that of SM-DO, suggesting that Sal-B, But, Tan IIA, and For are representative components of SM-DO for increasing VEGF levels. When only the VEGF level was taken as the response variable, the optimal proportion was 25% Sal-B: 60% But: 15% For, indicating that Sal-B, But, and For are the main components in OP for increasing the VEGF level. This is consistent with previous studies. For example, Nuan et al. (2020) reported that Sal-B could regulate the HIF-1α/VEGF pathway to promote angiogenesis in the myocardial tissue of MI rats, and For could promote the proliferation, migration, and tube formation of HUVECs by increasing VEGF expression (Liang et al., 2019).

The viability of endothelial cells is also a key factor in maintaining vascular integrity and promoting ischemic tissue repair (Martin-Bornez et al., 2023). The results showed that OP significantly increased the viability of OGD HCMVECs. The above results also showed concomitant changes in lactic acid and VEGF levels, suggesting that these indicators may be associated with the improvement in cell viability; however, the causal relationships have not been directly confirmed by experiments. When only viability was used as the response variable, the optimal proportion was 40% Sal-B: 29% But: 31% Tan IIA, suggesting that Sal-B, But, and Tan IIA are the main components in OP for improving the viability of OGD HCMVECs.

Under myocardial ischemia and hypoxia, cardiomyocytes in the infarcted area undergo loss of membrane integrity, increased permeability, and eventual rupture, causing cardiac enzymes such as cTnI, Myo, and CK-MB to leak into the bloodstream (Empana et al., 2022). HE staining reveals disordered myocardial arrangement, cellular rupture, and nuclear dissolution. Concurrently, excessive collagen fiber accumulation, as visualized by increased blue-stained collagen deposition in Masson staining, leads to ventricular remodeling, impaired systolic and diastolic function, and ultimately cardiac dysfunction (Zhu et al., 2022). The results showed that OP significantly reduced the cTnI level in OGD AC16 cells, but a certain deviation existed between the predicted and measured values. This may be because cTnI is a product of complex pathophysiological processes, and its release and clearance are regulated by multiple intrinsic mechanisms; angiogenesis is only one intervention pathway in this process. *In vivo* experimental results showed that OP significantly reduced serum levels of cTnI, Myo, and CK-MB in MI mice, ameliorated myocardial injury, attenuated myocardial fibrosis, and ultimately enhanced cardiac function in MI mice. These findings support the myocardial protective effect of OP at the whole-animal level. When only cTnI was taken as the response variable, the optimal proportion was 77% Sal-B: 23% For, suggesting that Sal-B and For are the main components in OP for reducing the cTnI level in OGD AC16 cells.

It is noteworthy that although OP was comparable to SM-DO in terms of lactic acid and VEGF levels, it was significantly weaker than SM-DO in improving HCMVEC viability and reducing cTnI. This may be because SM-DO contains other active components such as salvianolic acid A and cryptotanshinone. These components may act synergistically with the four components in OP through mechanisms such as promoting absorption and multi-target regulation, thereby enhancing the overall therapeutic efficacy. This also reflects the limitation of the “limited representative components” strategy—while the optimal proportion can retain some core pharmacological effects, it may lose non-core components with synergistic effects.

In addition, this study had a small sample size and no statistical power calculation; only male animals were used; and pharmacokinetic evaluation has not been conducted. Future studies will expand the sample size, include both male and female animals, incorporate more active components for mixture design to obtain a proportion combination with better efficacy and more comprehensive synergistic effects, and conduct pharmacokinetic and mechanistic validation experiments.

## 5 Conclusion

The results of this study showed that OP significantly promoted lactic acid production and upregulated VEGF expression in OGD HCMVECs, enhanced HCMVEC viability, and reduced cTnI release in OGD AC16 cells. *In vivo*, OP significantly increased myocardial levels of lactic acid and total protein lactylation, upregulated VEGF levels, reduced serum levels of cTnI, CK-MB, and Myo, alleviated myocardial tissue injury and fibrosis, and improved cardiac function in MI mice. These findings suggest that the four components can retain core pharmacological effects through scientific proportioning even without the complex system of the original herb. Compared with SM-DO, OP has clear components and an accurate proportion, providing a methodological reference for the application of mixture design in the study of active component compatibility of TCM. Future research may further advance the formulation development and preclinical research of this formula on the basis of completing relevant studies.

## Data Availability

The original contributions presented in the study are included in the article/Supplementary Material, further inquiries can be directed to the corresponding authors.
