# Supplementary material for: Mixture design optimization of salvianolic acid B, tanshinone IIA, butein, and formononetin from Salvia miltiorrhiza and Dalbergia odorifera for myocardial infarction
Source: Front Pharmacol. 2026 Jun 25;17:1783969. doi: 10.3389/fphar.2026.1783969 (PMC13345875; doi:10.3389/fphar.2026.1783969)
Supplement: Supplementary file 1 [file Table1.docx]

|  | | | | | | | | |
| --- | --- | --- | --- | --- | --- | --- | --- | --- |
| **No.** | **Sal-B** | **Butein** | **TanⅡA** | **For** | **HCMVECsviability（%）** | **HCMVECs lactic acid (mmol/gprot)** | **HCMVECs VEGF**  **(ng/gprot)** | **AC16 cells cTnI**  **(ng/mL)** |
| 1 | 1.000 | 0.000 | 0.000 | 0.000 | 0.50±0.02 | 0.58±0.06 | 51.52±3.87 | 5.87±0.61 |
| 2 | 0.000 | 1.000 | 0.000 | 0.000 | 0.50±0.03 | 0.61±0.16 | 57.97±7.25 | 6.92±0.58 |
| 3 | 0.000 | 0.000 | 1.000 | 0.000 | 0.47±0.03 | 0.59±0.10 | 61.79±4.27 | 5.23±0.41 |
| 4 | 0.000 | 0.000 | 0.000 | 1.000 | 0.54±0.03 | 1.28±0.14 | 86.92±5.52 | 8.01±0.72 |
| 5 | 0.500 | 0.500 | 0.000 | 0.000 | 0.56±0.03 | 0.93±0.15 | 73.54±7.93 | 6.46±0.83 |
| 6 | 0.500 | 0.000 | 0.500 | 0.000 | 0.62±0.01 | 1.01±0.17 | 67.10±5.28 | 5.71±0.59 |
| 7 | 0.500 | 0.000 | 0.000 | 0.500 | 0.62±0.03 | 0.47±0.16 | 51.62±3.98 | 5.39±0.63 |
| 8 | 0.000 | 0.500 | 0.500 | 0.000 | 0.63±0.03 | 0.55±0.08 | 63.68±4.91 | 7.20±0.45 |
| 9 | 0.000 | 0.500 | 0.000 | 0.500 | 0.60±0.03 | 0.78±0.12 | 55.05±3.53 | 8.92±0.74 |
| 10 | 0.000 | 0.000 | 0.500 | 0.500 | 0.65±0.04 | 1.03±0.04 | 91.77±1.90 | 6.09±0.85 |
| 11 | 0.333 | 0.333 | 0.333 | 0.000 | 0.72±0.03 | 0.64±0.06 | 41.40±7.85 | 6.45±0.90 |
| 12 | 0.333 | 0.333 | 0.000 | 0.333 | 0.58±0.02 | 0.69±0.08 | 55.04±7.32 | 4.56±0.77 |
| 13 | 0.333 | 0.000 | 0.333 | 0.333 | 0.54±0.03 | 0.48±0.09 | 59.78±8.53 | 6.12±0.62 |
| 14 | 0.000 | 0.333 | 0.333 | 0.333 | 0.57±0.02 | 0.86±0.11 | 54.07±7.02 | 7.56±0.73 |
| 15 | 0.250 | 0.250 | 0.250 | 0.250 | 0.61±0.02 | 0.84±0.16 | 71.13±6.26 | 4.64±0.84 |
| 16 | 0.625 | 0.125 | 0.125 | 0.125 | 0.60±0.03 | 0.67±0.06 | 90.98±7.61 | 7.76±0.87 |
| 17 | 0.125 | 0.625 | 0.125 | 0.125 | 0.61±0.04 | 0.81±0.10 | 91.08±3.81 | 7.15±0.70 |
| 18 | 0.125 | 0.125 | 0.625 | 0.125 | 0.60±0.02 | 0.65±0.07 | 66.57±4.92 | 5.59±0.88 |
| 19 | 0.125 | 0.125 | 0.125 | 0.625 | 0.59±0.03 | 0.31±0.10 | 38.51±3.85 | 7.33±0.75 |
