# Supplementary material for: Mixture design optimization of salvianolic acid B, tanshinone IIA, butein, and formononetin from Salvia miltiorrhiza and Dalbergia odorifera for myocardial infarction
Source: Front Pharmacol. 2026 Jun 25;17:1783969. doi: 10.3389/fphar.2026.1783969 (PMC13345875; doi:10.3389/fphar.2026.1783969)
Supplement: Supplementary file 2 [file Supplementaryfile3.docx]

**Figure S1. Response optimization plots.** (A–D) Response optimization plots for viability, vascular endothelial growth factor (VEGF), lactic acid, and cardiac troponin I (cTnI). The optimal proportion (OP) obtained from the mixture design was validated.

**Figure S2. Additional echocardiographic parameters.** (A–C) Left ventricular end‑systolic diameter (LVESD), left ventricular end‑systolic volume (LVESV), and left ventricular end‑diastolic volume (LVEDV) (means ± SD, n=6). ^##^*P*<0.01 vs. Sham; **P*<0.05, ***P*<0.01 vs. myocardial infarction (MI).
