# Supplementary material for: Mixture design optimization of salvianolic acid B, tanshinone IIA, butein, and formononetin from Salvia miltiorrhiza and Dalbergia odorifera for myocardial infarction
Source: Front Pharmacol. 2026 Jun 25;17:1783969. doi: 10.3389/fphar.2026.1783969 (PMC13345875; doi:10.3389/fphar.2026.1783969)
Supplement: Supplementary file 3 [file Table2.docx]

|  | | | | |
| --- | --- | --- | --- | --- |
| **Response variable** | **Regression equations** | **R^2^** | **Adjusted R^2^** | **Predicted R^2^** |
| HCMVECsviability | Y=0.5029X_1_+0.4977X_2_+0.4684X_3_+0.5366X_4_+0.2295X_1_X_2_+0.5552X_1_X_3_+0.3851X_1_X_4_+0.6009X_2_X_3_+0.3409X_2_X_4_+0.5728X_3_X_4_-0.447X_3_X_4_(X_3_-X_4_)-2.68X_1_^2^X_2_X_4-_7.51X_2_^2^X_3_X_4_+6.15X_1_X_2_^2^X_3_-10.24X_1_X_3_X_4_^2^ | 82.80% | 80.36% | 77.12% |
| VEGF | Y=51.74X_1_+59.42X_2_+63.01X_3_+87.13X_4_+68.5X_1_X_2_+38.9X_1_X_3_-74.6X_1_X_4_-76.2X_2_X_4_+66.8X_3_X_4_-254.5X_1_X_2_(X_1_-X_2_)-2315X_1_^2^X_2_X_3_-1195X_2_X_3_^2^X_4_-887X_1_X_3_X_4_^2^+2497X_1_X_2_(X_1_-X_2_)^2^-1644X_2_X_4_(X_2_-X_4_)^2^+5507X_1_X_2_X_3_X_4_ | 89.34% | 87.71% | 85.53% |
| lactic acid | Y=0.5794X_1_+0.5952X_2_+0.5726X_3_+1.2724X_4_+1.370X_1_X_2_+1.749X_1_X_3_-1.827X_1_X_4_-0.543X_2_X_4_+0.497X_3_X_4_-2.46X_1_X_2_(X_1_-X_2_)+8.78X_2_X_4_(X_2_-X_4_)-23.77X_1_X_2_^2^X_3_-31.25X_1_X_3_^2^X_4_-14.70X_2_X_4_(X_2_-X_4_)^2^+56.0X_1_X_2_X_3_X_4_ | 82.20% | 79.69% | 75.97% |
| cTnI | Y=5.929X_1_+6.928X_2_+5.282X_3_+8.016X_4_-6.33X_1_X_4_+4.19X_2_X_3_+5.73X_2_X_4_-2.34X_3_X_4_-21.77X_2_X_4_(X_2_-X_4_)+55.3X_1_^2^X_3_X_4_-189.0X_1_X_2_^2^X_4_+114.6X_1_X_2_(X_1_-X_2_)^2^-375.4X_1_X_2_X_3_X_4_ | 74.99% | 72.02% | 68.24% |
