# Supplementary material for: Mixture design optimization of salvianolic acid B, tanshinone IIA, butein, and formononetin from Salvia miltiorrhiza and Dalbergia odorifera for myocardial infarction
Source: Front Pharmacol. 2026 Jun 25;17:1783969. doi: 10.3389/fphar.2026.1783969 (PMC13345875; doi:10.3389/fphar.2026.1783969)
Supplement: Supplementary file 4 [file Table3.docx]

| **Response**  **variables** | **Measured**  **value** | **Predicted**  **value** | **Deviation**  **(%)** |
| --- | --- | --- | --- |
| HCMVECs  viability（%） | 0.60±0.05 | 0.60 | 0.00% |
| HCMVECs lactic acid (mmol/gprot) | 0.99±0.25 | 0.88 | 11.11% |
| HCMVECs VEGF (ng/gprot) | 92.86±10.30 | 102.88 | 10.12% |
| AC16 cTnI  (ng/mL) | 9.04±2.10 | 5.26 | 41.81% |
