# Supplementary figures and images for: Mixture design optimization of salvianolic acid B, tanshinone IIA, butein, and formononetin from Salvia miltiorrhiza and Dalbergia odorifera for myocardial infarction
Source: Front Pharmacol. 2026 Jun 25;17:1783969. doi: 10.3389/fphar.2026.1783969 (PMC13345875; doi:10.3389/fphar.2026.1783969)

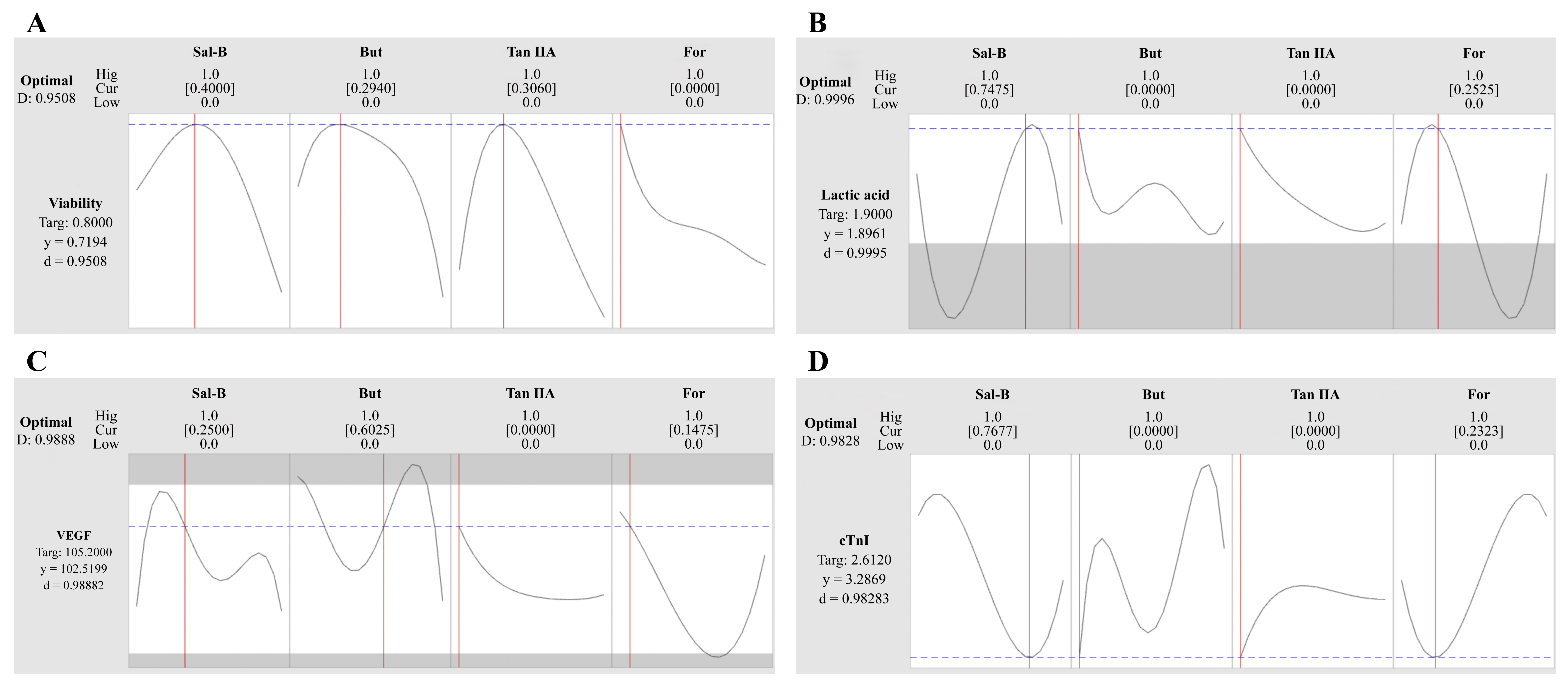

Supplement: Supplementary file 5 [file Supplementaryfile1.jpg]

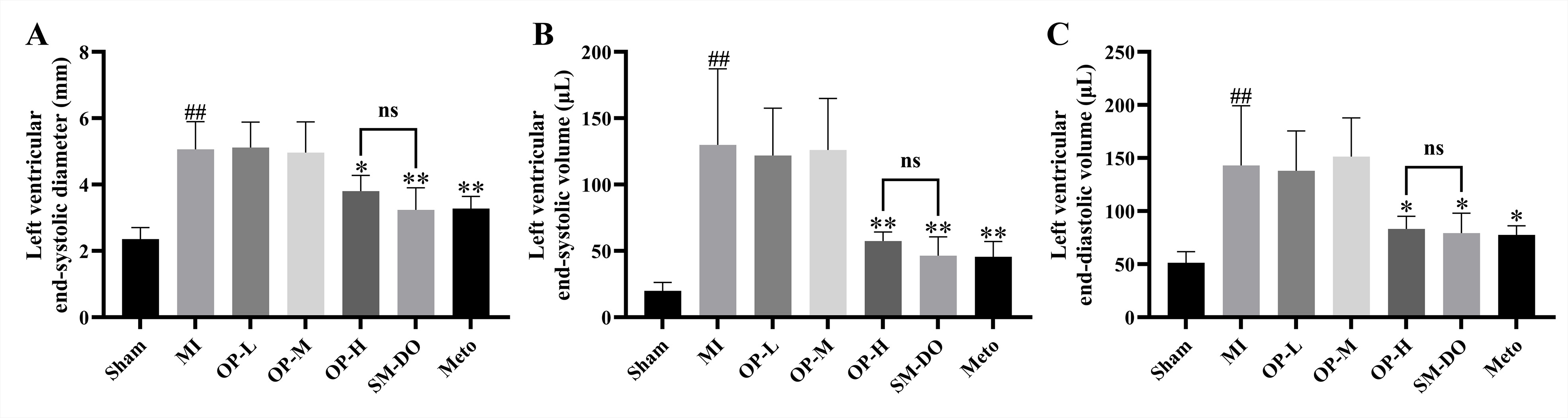

Supplement: Supplementary file 6 [file Supplementaryfile2.tif]
